# High‐throughput microfluidic real‐time PCR for the simultaneous detection of selected vector‐borne pathogens in dogs in Bosnia and Herzegovina

**DOI:** 10.1111/tbed.14645

**Published:** 2022-07-13

**Authors:** Vito Colella, Lucas Huggins, Adnan Hodžić, Clemence Galon, Rebecca Traub, Amer Alić, Roberta Iatta, Lénaïg Halos, Domenico Otranto, Muriel Vayssier‐Taussat, Sara Moutailler

**Affiliations:** ^1^ Department of Veterinary Medicine University of Bari Bari Apulia Italy; ^2^ Faculty of Veterinary and Agricultural Sciences University of Melbourne Melbourne Victoria Australia; ^3^ University of Veterinary Medicine Vienna Vienna Austria; ^4^ Ecole Nationale Vétérinaire d'Alfort Maisons‐Alfort France; ^5^ University of Sarajevo Sarajevo Bosnia and Herzegovina; ^6^ Bill & Melinda Gates Foundation Seattle Washington USA; ^7^ Bu‐Ali Sina University Hamedan Iran

**Keywords:** *Anaplasma*, Bosnia and Herzegovina, companion animals, *Leishmania*, microfluidic real‐time PCR, vector‐borne zoonoses

## Abstract

A scarcity of information on the occurrence of zoonotic vector‐borne pathogens (VBPs), alongside a lack of human and animal health authorities’ awareness of pre‐existing data, augment the risk of VBP infection for local people and limit our ability to establish control programs. This holds especially true in low‐middle income countries such as Bosnia and Herzegovina (BiH). This dearth of information on zoonotic VBPs is bolstered by the inability of previously used diagnostic tests, including conventional molecular diagnostic methods, to detect the full spectrum of relevant pathogens.

Considering this, we set out to apply a microfluidic qPCR assay capable of detecting 43 bacterial and protozoan pathogens from blood to accrue critical baseline data for VBPs occurrence in BiH. A total of 408 dogs were tested of which half were infected with at least one VBP of zoonotic or veterinary importance. *Leishmania infantum* was found in 18% of dogs, reaching a prevalence as high as 38% in urbanized areas of Sarajevo. These data highlight substantially higher levels of *L. infantum* prevalence when compared to that previously reported using conventional methods using the same samples. Additionally, this high‐throughput microfluidic qPCR assay was able to detect pathogens rarely or never reported in canines in BiH, including *Anaplasma phagocytophilum* (3%), *Anaplasma platys* (0.2%), haemotropic *Mycoplasma* (1%) and *Hepatozoon canis* (26%). Our report of the endemicity of important zoonotic pathogens and those of clinical significance to dogs emphasizes the need for urgent implementation of surveillance and control for VBPs in BiH, targeting both animal and human infections within the country.

## 1 INTRODUCTION

Canine vector‐borne pathogens (VBPs) encompass a broad diversity of disease‐causing organisms, that include viruses, bacteria, protozoa, and helminths predominantly unified by their ability to be transmitted via blood‐feeding arthropods, such as ticks, fleas, sandflies, and mosquitoes. Some VBPs generate severe disease solely in dogs, whilst others use canines as natural reservoir hosts of zoonotic agents thereby posing a risk to cohabiting humans as well (Otranto et al., 2017). To date, there has been very little information exploring the diversity and prevalence of canine VBPs in Bosnia and Herzegovina (BiH), with the few studies conducted thus far focusing on a limited number of key pathogen species, typically those of zoonotic significance (Ćoralić et al., 2018; Omeragić et al., 2018; Colella et al., 2019; Omeragić et al., 2021; Maksimović et al., 2022). These limited published studies have recorded high prevalence of canine VBP infection, exacerbated by a growing population of stray dogs since the end of the Bosnian war, that facilitates transmission and maintenance of VBPs in BiH (Ćoralić et al., 2018; Katica et al., 2019; Katica et al., 2020; Omeragić et al., 2021).

Prior investigations into canine VBPs in BiH have identified numerous zoonotic pathogens, in particular, a high prevalence of *Leishmania* spp. in dogs, alongside sporadic case reports of human infections (Alić et al., 2019; Colella et al., 2019; Obwaller et al., 2018; Vaselek, 2021). Human leishmaniases are among the most important VBPs globally, implicated in approximately 0.2–0.4 million human cases of visceral leishmaniasis (VL) and 0.7–1.2 million cases of cutaneous leishmaniasis (CL) annually (Alvar et al., 2012). A proportion of these cases are caused by *Leishmania infantum*, a pathogen that utilizes canine as reservoir hosts (Alvar et al., 2012; Dantas‐Torres, 2007; Okwor & Uzonna, 2016).

The tick species *Ixodes ricinus* and *Dermacentor reticulatus* are the most common ectoparasites infesting dogs in BiH (Krčmar et al., 2014; Omeragić, 2011). As a consequence, there have been reports of the bacterial VBP *Borrelia burgdorferi* sensu lato, which includes the aetiological agents of Lyme disease that can be harboured by canids (Arapović et al., 2014; Dautović‐Krkić et al., 2008), as well as the highly pathogenic, but not zoonotic, *Babesia canis* (Ćoralić et al., 2018). Additionally, the mosquito‐transmitted filarial worms *Dirofilaria immitis* and *Dirofilaria repens* have been reported in Bosnian dogs (Omeragić et al., 2018; Omeragić et al., 2021), with evidence of infection in humans caused by the latter zoonotic nematode (Omeragić et al., 2021; Zvorničanin et al., 2020).

Other canine VBPs that are prevalent in this country include the Oriental eyeworm *Thelazia callipaeda*, a zoonotic nematode parasite implicated in occasional human infections in Eastern Europe (Colella et al., 2016; Hodžić et al., 2014; Paradžik et al., 2016), and the apicomplexan pathogen *Hepatozoon canis* (Hodžić et al., 2015). However, there have been no reports to date of bacterial VBPs infecting Bosnian canines, likely due to a dearth of research given that evidence of such pathogens has been found in neighboring countries (Huber et al., 2017; Laušević et al., 2019). In addition, rickettsial pathogens have been molecularly detected in Bosnian ticks, including the zoonotic bacterial species *Anaplasma phagocytophilum* (Hodžić et al., 2017), the causative agent of potentially lethal human granulocytic anaplasmosis.

This dearth of information on zoonotic VBPs in BiH is caused by both the limited studies so far conducted and the inability of previously used diagnostic tests to detect the full spectrum of relevant pathogens. For example, conventional molecular methods such as endpoint PCR and real‐time PCR (qPCR) comprise some of the most sensitive and specific diagnostic methods available for the detection of VBPs, however, they can typically only detect a limited number of pathogens simultaneously and may require large volumes of template DNA per reaction (Gondard et al., 2018; Huggins et al., 2021; Michelet et al., 2014). More recently, advances in microfluidic technology have allowed for the development of microfluidic qPCR methods that employ microchips printed in chamber arrays as large as a 96 × 96, allowing for the simultaneous running of up to 9216 singleplex nanoliter quantity reactions (Gondard et al., 2020). The large number of reactions possible at any one time permits testing for numerous pathogens from multiple samples concurrently, reducing the amount of time, labour, and reagents used when compared to conventional qPCR, whilst also being able to easily identify coinfections. For example, microfluidic qPCR has been used to detect water‐borne and tick‐transmitted pathogens, permitting testing for as many as 37 organisms at the same time in various studies that have used it to unearth zoonoses as well as rare VBPs of veterinary importance (Gondard et al., 2020, 2018; Michelet et al., 2014; Sprong et al., 2019).

Given the significant paucity of research exploring VBPs and the potential zoonotic risk posed by some of them, we set out to use a microfluidic qPCR to test for 43 bacterial and protozoan pathogens and accrue baseline data for their occurrence in BiH. This is particularly timely given that economic and societal upheaval in BiH following the Bosnian war have had wide‐reaching impacts and may have potentially made the country vulnerable to an increase in neglected diseases such as those caused by VBPs.

## 2 MATERIALS AND METHODS

### 2.1 Sample collection and DNA extraction

**FIGURE 1:**
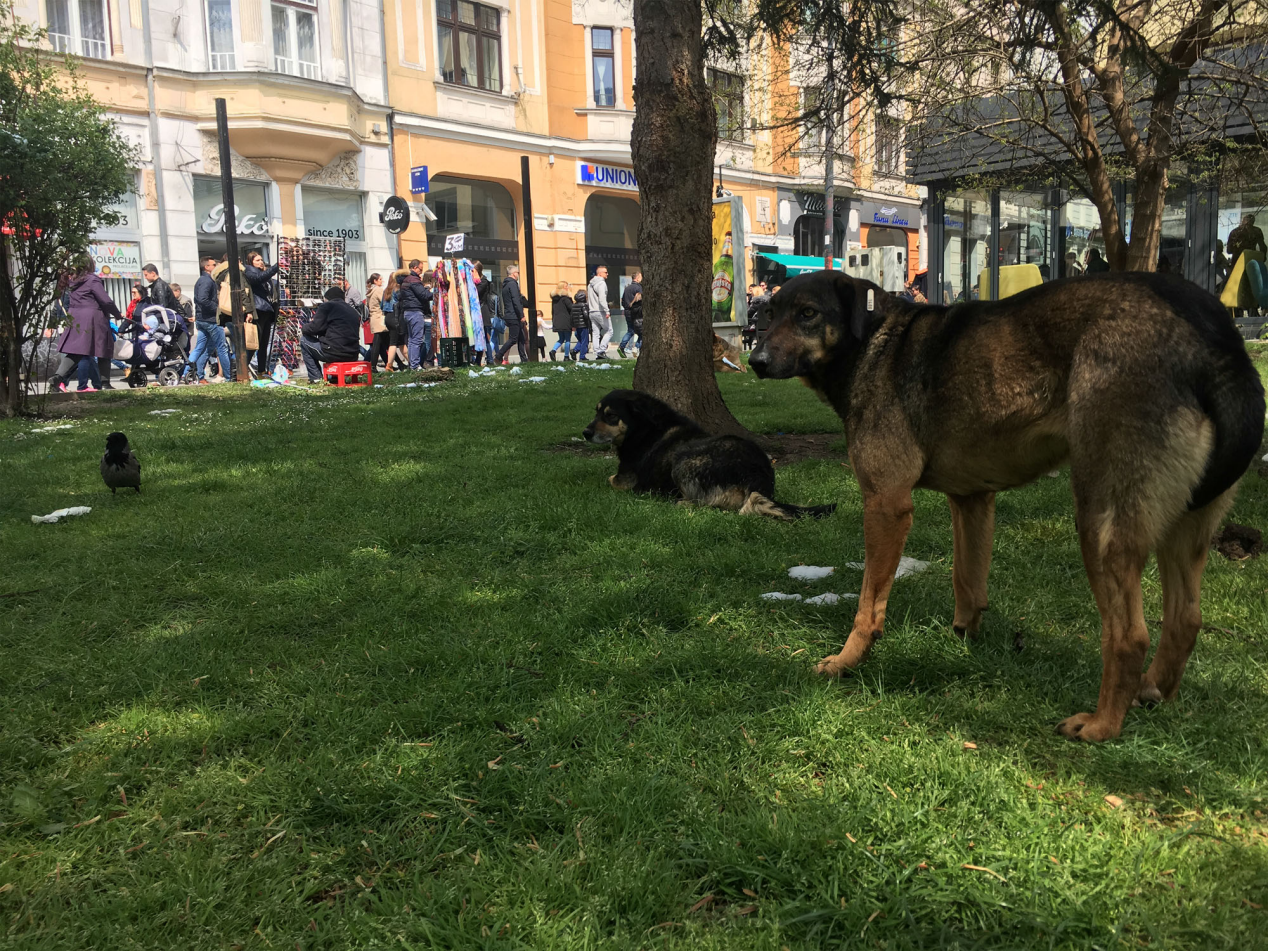
Free roaming dogs from a central urban area in Sarajevo, Bosnia and Herzegovina

In 2018, blood samples were collected from 408 domestic dogs kept under different living conditions (free‐roaming [*n* = 127], client‐owned (*n* = 134), sheltered [*n* = 142] and not reported [*n* = 4]) from different urban localities of BiH, namely Sarajevo (*n* = 138), Gračanica (*n* = 63), Zenica (*n* = 44), Goražde (*n* = 40), Bihać (*n* = 40), Mostar (*n* = 35), Livno (*n* = 14), Gornji Vakuf‐Uskoplje (*n* = 12), Odžak (*n* = 11) and Tuzla (*n* = 10) (Figure 1). Two millilitres of whole blood were collected per dog by a veterinarian via cephalic or jugular puncture into an anti‐coagulation ethylenediaminetetraacetic acid (EDTA) tube and stored at − 20°C until further analysis. Genomic DNA was extracted from these samples using the GenUP DNA Kit (Biotechrabbit, Germany), following the manufacturer's recommendations.

### 2.2 DNA pre‐amplification

All DNA samples were subjected to pre‐amplification to enrich DNA content compared to host DNA using the PerfeCTa PreAmp SuperMix (Quanta Biosciences, Beverly, USA), following the manufacturer's guidelines. All the primers were pooled (except positive control *Escherichia coli* culture EDL 933 strain), with a final and equal concentration of 200 nM each. The pre‐amplification reaction was performed in a final volume of 5 μl containing 1 μl Perfecta Preamp 5×, 1.25 μl pooled primers mix, 1.5 μl distilled water and 1.25 μl DNA. PCR cycling conditions were one cycle at 95°C for 2 min followed by 14 cycles at 95°C for 10 s and 60°C for 3 min. At the end of the cycling program, the reactions were diluted at 1:10 and stored at – 20°C until further use.

### 2.3 High‐throughput microfluidic real‐time PCR

The BioMark real‐time PCR system (Fluidigm, USA) was used for high‐throughput microfluidic real‐time PCR amplification using 48.48 dynamic arrays (Fluidigm, USA). These chips dispensed 48 samples and 48 assays into individual wells, followed by on‐chip real‐time PCR reactions in individual chambers and thermal cycling, resulting in 2,304 individual reactions as reported in Michelet et al., 2014. Targeted microorganisms were *Borrelia* spp., *Borrelia burgdorferi* sensu stricto, *Borrelia garinii*, *Borrelia afzelii*, *Borrelia valaisiana*, *Borrelia lusitaniae*, *Borrelia spielmanii*, *Borrelia bissettii*, *Borrelia miyamotoi*, *Anaplasma* spp., *Anaplasma marginale*, *Anaplasma platys*, *Anaplasma phagocytophilum*, *Anaplasma ovis*, *Anaplasma centrale*, *Anaplasma bovis*, *Ehrlichia* spp., *Ehrlichia canis*, *Neoehrlichia mikurensis*, *Rickettsia* spp., *Rickettsia conorii*, *Rickettsia slovaca*, *Rickettsia massiliae*, *Rickettsia helvetica*, *Rickettsia aeschlimannii*, *Rickettsia felis*, *Bartonella* spp., *Bartonella henselae*, *Francisella* spp., *Coxiella* spp., Apicomplexa, *Babesia microti*, *Babesia vogeli*, *Babesia ovis*, *Babesia bovis*, *Babesia caballi*, *Babesia venatorum*, *Babesia divergens*, *Mycoplasma* spp., *Theileria* spp., *Hepatozoon* spp., *Leishmania* spp. and *Leishmania infantum*. Targeted genes and primers/probes set for the pathogens above listed are reported in Gondard et al., 2020; Michelet et al., 2014; Sprong et al., 2019.

Briefly, amplifications were performed using 6‐carboxyfluorescein (FAM)‐ and black hole quencher (BHQ1)‐labelled TaqMan probes with PerfeCTa qPCR ToughMix, Low ROX (QuantaBio) following a previously described protocol (Michelet et al., 2014). Two kinds of controls per chip were used for experimental validation: a negative water control to exclude contamination and internal control, to check for the presence of qPCR reaction inhibitors comprising *Escherichia coli* strain EDL933 DNA and relevant primers and probe to detect this (Gondard et al., 2020). Data were acquired on the BioMark Real‐TimePCR System and analyzed using the Fluidigm Real‐time PCR Analysis Software to obtain crossing point (CP) values. Each sample was run in duplicate and considered positive only when both reactions displayed a CP value. Samples with a CP value > 30 were considered negatives (cut off value).

To ascertain the species of pathogens for which only targets at genus level were detectable via microfluidic real‐time PCR (i.e., haemotropic *Mycoplasma* spp.*, Babesia* spp., and *Hepatozoon* spp.), selected positive samples for the above‐mentioned genera were also tested via conventional and nested PCRs using primers listed in Table 1. Amplicons were sequenced in both directions by LGC Genomics, Germany, using Sanger sequencing and nucleotide sequences were assembled using the software BioEdit (www.mbio.ncsu.edu/BioEdit/bioedit.html). An online BLAST (National Center for Biotechnology Information) was used to compare results with published sequences listed in the GenBank sequence databases.

**TABLE 1:** Target organism, gene target, and primer sequences used in this study to confirm vector‐borne pathogens detected by microfluidic qPCR

**Target organism**	**Gene target**	**Primer sequences (5´→ 3´)**	**Product size (bp)**	**Reference**
Apicomplexa *Babesia / Theileria*	18S rRNA	**BTH‐1F**: CCTGAGAAACGGCTACCACATCT **BTH‐1R**: TTGCGACCATACTCCCCCCA Nested **GF2**: GTCTTGTAATTGGAATGATGG **GR2**: CCAAAGACTTTGATTTCTCTC	720 / 750 590 / 610	(52)
*Hepatozoon* spp.	18S rRNA	**H14Hepa18SFw**: GAAATAACAATACAAGGCAGTAAAATGCT **H14Hepa18SRv**: GTGCTGAAGGAGTCGTTTATAAAGA	620	(21)
Haemotropic *Mycoplasma*	16S rRNA	**HBT‐F**: TACGGCCCATATTCCTACG **HBT‐R**: TGCTCCACCACTTGTTCA	600	(53)

### 2.4 Statistical analysis

95% confidence intervals (CIs) for proportions were calculated using the Wilson score interval via the open‐source software Epitools (https://epitools.ausvet.com.au). Multiple logistic regression analysis was used to infer canine risk factors such as sex, age, and husbandry (shelter, owned, and stray) for VBPs infections in Prism 9 (San Diego, CA, USA). A strategy of iterative backward elimination was used to arrive at final models. In each iteration, the explanatory variable that had the largest *p*‐value of those exceeding a threshold α* = *0.2 was removed and a new model was fitted. Associations between explanatory variables and the response variable were considered statistically significant if their *p* < .05. Pearson's Chi‐squared test of independence was used in the case of a single categorical explanatory variable.

## 3 RESULTS

**FIGURE 2:**
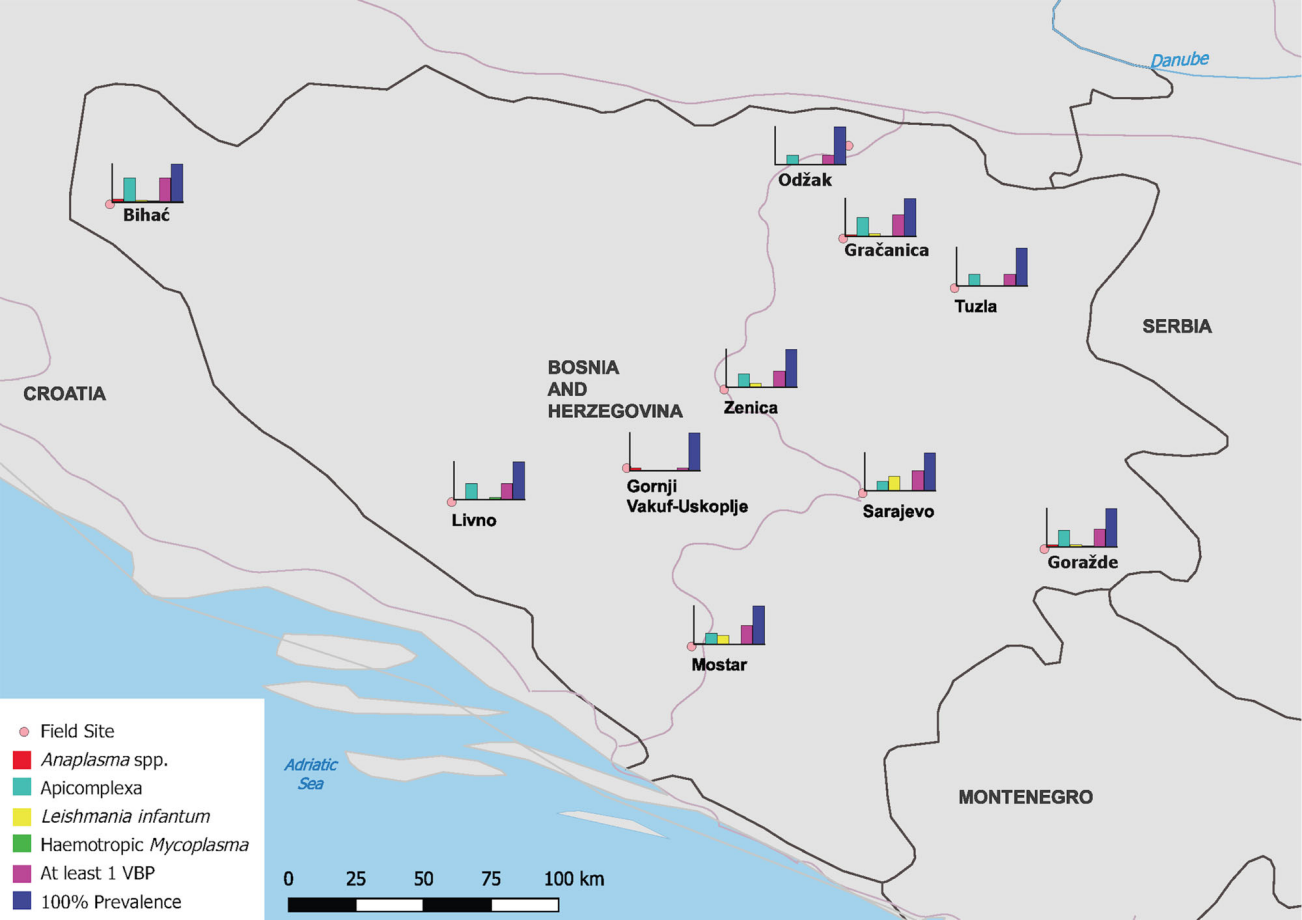
Map of field sites sampled over the course of this study in Bosnia and Herzegovina with prevalence for *Anaplasma* spp., Apicomplexa, *Leishmania infantum*, haemotropic *Mycoplasma*, and positivity to at least one vector‐borne pathogen (VBP). Map created in QGIS 3.4 via QGIS.org, 2021. QGIS Geographic Information System. QGIS Association

Positive controls were correctly identified in each cycle using their corresponding set of primers and probes. Of the 408 dogs sampled and tested, 204 (50%; CI: 45–55%) were found positive for at least one VBP across all field sites investigated (Figure 2, Table 2). The estimated prevalence of VBP infection in dogs with at least one pathogen ranged from 8% (CI: 1–35%) in Gornji Vakuf‐Uskoplje to 57% (CI: 45–69%) and 63% (CI: 47–76%) in Gračanica and Bihać, respectively (Figure 2). The most frequently identified target was Apicomplexa in 141 dogs (35%; CI: 30– 39%), followed by *Hepatozoon* spp. in 107 dogs (26%; CI: 22–31%) and *L. infantum* in 74 dogs (18%; CI: 15–22%) (Table 2). Additionally, the zoonotic bacterial VBP *A. phagocytophilum* was detected in 11 canines (3%; CI: 2–5%), with peaks of 8% in Bihać and Gornji Vakuf‐Uskoplje (Table 2).

**TABLE 2:** Number of positive dogs and pathogen prevalence with 95% confidence intervals found for vector‐borne pathogen infections in 408 dogs from ten different field sites in Bosnia and Herzegovina, as detected by microfluidic qPCR

	**Vector‐Borne Pathogens**							
**Field site**	***A. platys***	***A. phagocytophilum***	**Apicomplexa**	***B. vogeli***	***Hepatozoon* spp**.	***L. infantum***	**Haemotropic *Mycoplasma***	**At least 1 VBP**
**Sarajevo (*n* = 138)**	0 (0%; 0–3%)	2 (1%; 0–5%)	36 (26%; 19–34%)	2 (1%; 0–5%)	17 (12%; 8–19%)	52 (38%; 30–46%)	1 (0%; 0–4%)	75 (54%; 46–62%)
**Gračanica (*n* = 63)**	0 (0%; 0–6%)	3 (5%; 2–13%)	30 (48%; 36–60%)	0 (0%; 0–6%)	18 (29%; 19–41%)	5 (8%; 3–17%)	2 (3%; 0–11%)	36 (57%; 45–69%)
**Tuzla (*n* = 10)**	0 (0%; 0–28%)	0 (0%; 0–28%)	3 (30%; 11–60%)	0 (0%; 0–28%)	2 (20%; 6–51%)	0 (0%; 0–28%)	0 (0%; 0–28%)	3 (30%; 11–60%)
**Mostar (*n* = 35)**	1 (3%; 1–15%)	0 (0%; 0–10%)	9 (26%; 14–42%)	0 (0%; 0–10%)	10 (29%; 16–45%)	8 (23%; 12–39%)	0 (0%; 0–10%)	17 (49%; 33–64%)
**Livno (*n* = 14)**	0 (0%; 0–22%)	0 (0%; 0–22%)	5 (35%; 16–61%)	0 (0%; 0–22%)	4 (29%; 12–55%)	0 (0%; 0–22%)	1 (7%; 1–31%)	6 (43%; 21–67%)
**Goražde (*n* = 40)**	0 (0%; 0–9%)	2 (5%; 1–17%)	14 (35%; 22–50%)	0 (0%; 0–9%)	17 (43%; 29–58%)	2 (5%; 1–17%)	1 (3%; 0–13%)	19 (47%; 33–63%)
**Odžak (*n* = 12)**	0 (0%; 0–24%)	0 (0%; 0–24%)	3 (25%; 9–53%)	0 (0%; 0–24%)	1 (8%; 1–35%)	0 (0%; 0–24%)	0 (0%; 0–24%)	3 (25%; 9–53%)
**G. Vakuf (*n* = 12)**	0 (0%; 0–24%)	1 (8%; 1–35%)	0 (0%; 0–24%)	0 (0%; 0–24%)	0 (0%; 0–24%)	0 (0%; 0–24%)	0 (0%; 0–24%)	1 (8%; 1–35%)
**Zenica (*n* = 44)**	0 (0%; 0–8%)	0 (0%; 0–8%)	16 (36%; 24–51%)	0 (0%; 0–8%)	14 (32%; 20–47%)	5 (11%; 5–24%)	0 (0%; 0–8%)	19 (43%; 30–58%)
**Bihać (*n* = 40)**	0 (0%; 0–9%)	3 (8%; 3–20%)	25 (63%; 47–76%)	1 (3%; 0–13%)	24 (60%; 45–74%)	2 (5%; 1–17%)	1 (3%; 0–13%)	25 (63%; 47–76%)
**All Sites (*n* = 408)**	1 (0.2%; 0–1%)	11 (3%; 2–5%)	141 (35%; 30–39%)	3 (0.7%; 0–2%)	107 (26%; 22–31%)	74 (18%; 15–22%)	6 (1%; 0.6–3%)	204 (50%; 45–55%)

Coinfections by two pathogens were diagnosed in 36 dogs (9%; CI: 6–12%), with the most common being *H. canis* with *L. infantum* (*n* = 16 dogs; 4%; CI: 2–6%), followed by *A. phagocytophilum* with *H. canis* (*n* = 7 dogs; 2%; CI: 1– 4%) and Apicomplexa with *L. infantum* (*n* = 7 dogs; 2%; CI: 1–4%) (Table 3).

**TABLE 3:** Number of positive dogs and pathogen prevalence with 95% confidence intervals found for vector‐borne pathogen coinfections in 408 dogs from ten different field sites in Bosnia & Herzegovina, as detected by microfluidic qPCR

	**Vector‐Borne Pathogen Coinfections**					
**Field Site**	***A. phagocytophilum* & *H. canis***	**Apicomplexa & *L. infantum***	***B. vogeli* & *H. canis***	**Apicomplexa & *Mycoplasma***	***H. canis* & *L. infantum***	***H. canis* & *Mycoplasma***
**Sarajevo (*n *= 138)**	2 (1%; 0–5%)	6 (4%; 2–9%)	0 (0%; 0–3%)	1 (0%; 0–4%)	7 (5%; 2–10%)	0 (0%; 0–3%)
**Gračanica (*n* = 63)**	1 (2%; 0–8%)	1 (2%; 0–8%)	0 (0%; 0–6%)	1 (2%; 0–8%)	2 (3%; 0–11%)	0 (0%; 0–6%)
**Tuzla (*n* = 10)**	0 (0%; 0–28%)	0 (0%; 0–28%)	0 (0%; 0–28%)	0 (0%; 0–28%)	0 (0%; 0–28%)	0 (0%; 0–28%)
**Mostar (*n* = 35)**	0 (0%; 0–10%)	0 (0%; 0–10%)	0 (0%; 0–10%)	0 (0%; 0–10%)	2 (6%; 2–19%)	0 (0%; 0–10%)
**Livno (*n* = 14)**	0 (0%; 0–22%)	0 (0%; 0–22%)	0 (0%; 0–22%)	1 (7%; 1–31%)	0 (0%; 0–22%)	0 (0%; 0–22%)
**Goražde (*n* = 40)**	1 (3%; 0–13%)	0 (0%; 0–9%)	0 (0%; 0–9%)	0 (0%; 0–9%)	1 (3%; 0–13%)	1 (3%; 0–13%)
**Odžak (*n* = 12)**	0 (0%; 0–24%)	0 (0%; 0–24%)	0 (0%; 0–24%)	0 (0%; 0–24%)	0 (0%; 0–24%)	0 (0%; 0–24%)
**G. Vakuf (*n* = 12)**	0 (0%; 0–24%)	0 (0%; 0–24%)	0 (0%; 0–24%)	0 (0%; 0–24%)	0 (0%; 0–24%)	0 (0%; 0–24%)
**Zenica (*n* = 44)**	0 (0%; 0–8%)	0 (0%; 0–8%)	0 (0%; 0–8%)	0 (0%; 0–8%)	2 (5%; 1–15%)	0 (0%; 0–8%)
**Bihać (*n* = 40)**	3 (8%; 3–20%)	0 (0%; 0–9%)	1 (3%; 0–13%)	0 (0%; 0–9%)	2 (5%; 1–17%)	1 (3%; 0–13%)
**All Sites (*n* = 408)**	7 (2%; 1–4%)	7 (2%; 1–4%)	1 (0.2%; 0–1%)	3 (0.7%; 0–2%)	16 (4%; 2–6%)	2 (0.5%; 0–2%)

For the samples found positive by microfluidic real‐time PCR that could not be characterized to species level for example samples found positive to Apicomplexa, *Hepatozoon* spp. and haemotropic *Mycoplasma* we used cPCR and Sanger sequencing to gain species‐level resolution. This allowed us to detect *Babesia canis*, *H. canis*, *Mycoplasma haematoparvum* and *Mycoplasma haemocanis* with all sequences showing 100% identity with reference sequences. These sequences have been deposited in GenBank under accession numbers MK107800‐MK107818).

No model could be fitted with predictor variables for positivity to at least one VBP. However, we found evidence of an association between owned [OR 2.26 (95% CI: 1.17–4.5), *p* = 0.01] and stray [OR 2.23 (95% CI: 1.15–4.45), *p* = 0.02] dogs compared to sheltered animals on the odds of being infected by *L. infantum*.

## 4 DISCUSSION

The deployment of a high‐throughput microfluidic qPCR method has elucidated a spectrum of VBPs that Bosnian canines are infected with, including those that are zoonotic, with at least half of all dogs sampled infected with one VBP. In addition, this research has unearthed the presence of VBPs (e.g., *A. phagocytophilum*, *A. platys*, haemotropic mycoplasmas and *H. canis*) rarely or never reported in Bosnian dogs, therefore expanding the known geographical distributions of these pathogens.

With the aid of this highly sensitive technique, we were able to detect the zoonotic VBP *L. infantum* in 18% of dogs sampled across BiH, with a particularly high prevalence around urbanized areas of BiH, such as Sarajevo (38%) and Mostar (23%). These data highlight substantially higher levels of *L. infantum* prevalence when compared to that previously reported (Colella et al., 2019). Nonetheless, this study found similar foci for *L. infantum* infection to the data presented here, with prevalence highest in Mostar (16.7%), followed by Sarajevo (4.9%) (Colella et al., 2019).

*Leishmania infantum* is a causative agent of VL and CL in humans with prior research having an estimated annual incidence of 2–3 cases per 100,000 in BiH (Alvar et al., 2012), alongside occasional case reports (Gvozdenović & Miladinović, 1959; Obwaller et al., 2018). Our identification of *L. infantum* in dogs in BiH is made even more pertinent given that when national institutes and ministries in the country were surveyed regarding the presence of endemic animal leishmaniosis, this disease was not known to exist, despite recent research demonstrating it to be endemic (Berriatua et al., 2021; Colella et al., 2019). Given that autochthonous cases of animal leishmaniasis are notifiable in BiH, it would appear that contemporary data on *L. infantum* prevalence may not be effectively communicated to local ministries and veterinarians, resulting in cases of animal leishmaniosis potentially being undiagnosed and unreported (Berriatua et al., 2021). Substantial efforts should be made to keep local veterinarians and clinicians abreast of such findings regarding the prevalence of *L. infantum* in BiH due to the substantial risk this pathogen poses to humans and animals in the country (Colella et al., 2019). Additionally, our research identified *L. infantum* in concomitant infections, for example with *H. canis* in 4% of dogs, a finding that has not previously been made in BiH and may have pathological implications.

The identification of dogs infected with *A. phagocytophilum* is a key finding given that dogs act as sentinel hosts for this important zoonotic pathogen, the aetiology of granulocytic anaplasmosis, which is a potentially lethal disease in humans (Carrade et al., 2009; Ganta, 2021). *Anaplasma phagocytophilum* infects canines as accidental hosts in which the disease is usually self‐limiting, exhibited through clinical signs such as temporary lethargy, fever, inappetence, and lameness (Carrade et al., 2009). This pathogen has been only recently reported from dogs in BiH (Maksimović et al., 2022), although molecularly identified in Bosnian ticks in 2017 (Hodžić et al., 2017), other than being highly prevalent in ticks from Serbia, which shares an eastern border with BiH (Milutinović et al., 2008). This may be due to the high circulation of *I. ricinus* in the investigated area as well as to the role of other wild canids (e.g., foxes) as reservoirs for this pathogen species (Sgroi et al., 2021).

The apicomplexan species *H. canis* and the highly pathogenic *B. canis* were identified at a high prevalence in sampled dogs from BiH. *Babesia canis* is the most common species detected in dogs across Europe, causing anorexia and lethargy in its initial stage, that can later progress to clinical signs including fever, thrombocytopenia, and anaemia and can prove fatal (Ćoralić et al., 2018; Furlanello et al., 2005). This *Babesia* species has been previously identified in as many as 82.5% of dogs in BiH (Ćoralić et al., 2018) as well as in the neighbouring countries of Croatia and Serbia (Brkljačić et al., 2010; Davitkov et al., 2015). This study firstly reports *H. canis* in dogs in BiH, with this species comprising the most prevalent VBP detected in our cohort of sampled dogs, and the most common coinfection alongside *L. infantum*. *Hepatozoon canis* is transmitted to dogs by the ingestion of the tick vector as opposed to ectoparasite blood feeding and often causes a subclinical infection, although in some scenarios, for example in immunosuppressed hosts, it can generate disease (Baneth et al., 2003; Hodžić et al., 2015; Huggins et al., 2019). This VBP has been previously identified in 38.6% of red foxes from BiH (Hodžić et al., 2015) and less commonly by molecular means in dogs sampled in Serbia and Croatia (Gabrielli et al., 2015; Vojta et al., 2009). Unexpectedly, pathogens vectored by *Rhipicephalus sanguineus* sensu lato, such as *A. platys* and *H. canis*, were detected in dog population herein examined but not *E. canis*, despite its presence in dogs from neighbouring countries such as Montenegro (Laušević et al., 2019).

Our identification of the haemotropic *M. haematoparvum* and *M. haemocanis* in Bosnian dogs, as well as *A. platys*, is also novel data that extends the known endemic geographical range of these canine VBPs. Although none of these pathogen species is associated with significant lethality in dogs, *A. platys* is the aetiological agent of infectious canine cyclic thrombocytopaenia, a disease known to exacerbate clinical signs in dogs when identified in the context of VBP coinfections (Gaunt et al., 2010; Huggins et al., 2022). In addition, haemotropic *Mycoplasma* spp. can also produce mild disease in canines, however, such disease may be magnified in scenarios where the host is immunocompromized, potentially causing severe anaemia, leukopaenia, and cachexia (Compton et al., 2012). Similarly, co‐infection with *A. platys, Bartonella henselae* and *Mycoplasma haematoparvum* seems to worsen clinical presentations in infected human patients (Maggi et al., 2013).

The high‐throughput microfluidic qPCR assay applied herein is capable of detecting a large panel of agents and was instrumental for the identification of VBPs previously unreported in Bosnian canines, including species that may have been missed by conventional molecular methods. Typically, when utilizing cPCR or qPCR assays to conduct epidemiological surveys, prior data on pathogen prevalence in the region must be used to inform the selection of diagnostic targets, as each may only be able to detect one or a few pathogens. Therefore, in the context of regions with scarce previous epidemiological data selection of such conventional diagnostics may be suboptimal and pathogens missed (Gondard et al., 2020; Huggins et al., 2020). Moreover, our microfluidic qPCR's pre‐amplification step, which has previously been demonstrated to greatly improve the sensitivity of this assay, may have permitted enhanced detection capability of the VBPs herein detected (Gondard et al., 2020; Michelet et al., 2014). This improved sensitivity may provide us with more accurate estimated prevalence data, particularly given that these VBPs usually circulate at low parasite DNA concentrations or generate transient parasitaemia in dogs (de Caprariis et al., 2011; Oliva et al., 2006; Paradies et al., 2010). For example, our pre‐amplification step in this study may explain the increased detection of *L. infantum* in approximately 15% more dogs than the percentage of positives elucidated before (Colella et al., 2019).

Overall, the microfluidic qPCR method used in this study may be highly suited to large‐scale epidemiological surveys of canine VBPs, particularly in countries with scarce prior data in conjunction with large free‐roaming dog populations that may facilitate easy and unbridled VBP transmission (Katica et al., 2020; Omeragić et al., 2021). The large number of samples that can be screened concurrently for numerous VBP species, makes microfluidic qPCR techniques amenable to surveys that rely on a high‐throughput diagnostic capability (Gondard et al., 2020; Michelet et al., 2014). Nonetheless, cross‐validation might be needed for the characterization of some VBPs to a species level that is, through the use of conventional PCR and Sanger sequencing, which rules the microfluidic method less suited to diagnosis in a clinical setting, for which more specific and bespoke molecular or serological tests may be available.

Here our report of endemicity of the highly pathogenic and zoonotic pathogens *L. infantum* and *A. phagocytophilum* alongside a lack of awareness by the relevant animal and human health authorities regarding the occurrence of these pathogens in BiH, emphasizes the urgent need for allocation of resources to implement surveillance and control, targeting both animal and human infections within the country.

## CONFLICT OF INTEREST

The authors declare no conflict of interest.

## ETHICS STATEMENT

The authors confirm that the ethical policies of the journal, as noted on the journal's author guidelines page, have been adhered to. The study was performed in accordance with the Veterinary law of Bosnia and Herzegovina (“OJ BiH”, no: 34/02), the Game law of Bosnia and Herzegovina (“OJ BiH”, no: 4/06), and the Annual Program of Measures for Animal Health Protection in Bosnia and Herzegovina (“OJ BiH”, no: 15/18).

## Data Availability

The data that support the findings of this study are available from the corresponding author upon reasonable request.
